# Effects of gamma radiation on engineered tomato biofortified for space agriculture by morphometry and fluorescence-based indices

**DOI:** 10.3389/fpls.2023.1266199

**Published:** 2023-10-09

**Authors:** Riccardo Pagliarello, Elisabetta Bennici, Ilaria Di Sarcina, Maria Elena Villani, Angiola Desiderio, Luca Nardi, Eugenio Benvenuto, Alessia Cemmi, Silvia Massa

**Affiliations:** ^1^ Biotechnology Laboratory, Biotechnology and Agro-Industry Division, Department for Sustainability, Casaccia Research Center, Italian National Agency for New Technologies, Energy and Sustainable Economic Development (ENEA), Rome, Italy; ^2^ Department of Agriculture and Forest Sciences (DAFNE), University of Tuscia, Viterbo, Italy; ^3^ Fusion and Nuclear Safety Technologies Department, Casaccia Research Center, Italian National Agency for New Technologies, Energy and Sustainable Economic Development (ENEA), Rome, Italy

**Keywords:** gamma rays, space environment simulation, agrospace, biofortified MicroTom, morphometric and fluorimetric analysis

## Abstract

**Introduction:**

Future long-term space missions will focus to the solar system exploration, with the Moon and Mars as leading goals. Plant cultivation will provide fresh food as a healthy supplement to astronauts’ diet in confined and unhealthy outposts. Ionizing radiation (IR) are a main hazard in outer space for their capacity to generate oxidative stress and DNA damage. IR is a crucial issue not only for human survival, but also for plant development and related value-added fresh food harvest. To this end, efforts to figure out how biofortification of plants with antioxidant metabolites (such as anthocyanins) may contribute to improve their performances in space outposts are needed.

**Methods:**

MicroTom plants genetically engineered to express the *Petunia hybrida PhAN4* gene, restoring the biosynthesis of anthocyanins in tomato, were used. Seeds and plants from wild type and engineered lines AN4-M and AN4-P_2_ were exposed to IR doses that they may experience during a long-term space mission, simulated through the administration of gamma radiation. Plant response was continuously evaluated along life cycle by a non-disturbing/non-destructive monitoring of biometric and multiparametric fluorescence-based indices at both phenotypic and phenological levels, and indirectly measuring changes occurring at the primary and secondary metabolism level.

**Results:**

Responses to gamma radiation were influenced by the phenological stage, dose and genotype. Wild type and engineered plants did not complete a seed-to-seed cycle under the exceptional condition of 30 Gy absorbed dose, but were able to cope with 0.5 and 5 Gy producing fruits and vital seeds. In particular, the AN4-M seeds and plants showed advantages over wild type: negligible variation of fluorimetric parameters related to primary metabolism, no alteration or improvement of yield traits at maturity while maintaining smaller habitus than wild type, biosynthesis of anthocyanins and maintained levels of these compounds compared to non-irradiated controls of the same age.

**Discussion:**

These findings may be useful in understanding phenotypic effects of IR on plant growth in space, and lead to the exploitation of new breeding efforts to optimize plant performances to develop appropriate ideotypes for future long-term space exploration extending the potential of plants to serve as high-value product source.

## 1 Introduction

Human space colonization will rely on artificial environments where higher plants have an essential role in releasing oxygen, absorbing carbon dioxide, purifying water and producing fresh food for feeding crews (Fu et al., 2016; Zabel et al., 2016; Braun et al., 2018). An ideal space plant would cope with such artificial environments in extra-terrestrial conditions to be a valuable source of fresh food and functional compounds necessary for the astronauts’ diet, as well (Hessel et al., 2022).

Higher plants cultivation in space must specially consider ionizing radiation (IR). Measurements aboard the Apollo lunar flights are the only existing data from crewed spacecraft outside Earth magnetosphere. Mean dose rates of the Apollo missions ranged from 220 to 1,270 µGy/day (Benton and Benton, 2001). Recently, the LND (Lunar Lander Neutrons and Dosimetry) aboard the Chang’E 4 and the CRaTER (Cosmic Ray Telescope for the Effects of Radiation) aboard the LRO (Lunar Reconnaissance Orbiter) recorded the Moon radiation environment as 10 and 13.2 µGy/hour, respectively. Therefore, the estimated Galactic Cosmic Rays (GCR) exposure for lightly shielded environments on the lunar surface has been calculated as about 200 µGy/day during solar minimum conditions (Zhang et al., 2020). Conversely, the exposure to radiation during interplanetary cruise to Mars has been estimated using models of different type such as GEANT4, HZETRN, OLTARIS and PHITS and using radiation data obtained MARIE, GOES-8 and RAD (Radiation Assessment Detector). To date, based on data obtained from RAD on board the Mars Science Laboratory Rover Curiosity, is estimated that, during a 680-day mission (i.e., 180-day journey and 500-day stay on Martian surface), a living organism is exposed to approximately 1.01 Gy (Hu et al., 2009; Hassler et al., 2014; Guo et al., 2017; Chancellor et al., 2018).

As components of cosmic IR, gamma-rays can damage DNA both directly, by introducing double stranded breaks, and indirectly *via* radiolysis, which creates reactive oxygen species (ROS), such as hydrogen peroxide, superoxide anion, hydroxyl radicals and singlet oxygen (Gudkov et al., 2019). IR demonstrated to affect morpho-anatomical traits (e.g., cell wall structure, growth rate, reproductive success), nucleic acids, proteins and the photosynthetic system, with consequences also on food quality (De Pascale et al., 2021). Furthermore, plant age and time of exposure may play an important role in integrating radiation effects (Kurimoto et al., 2010). The IR damage to DNA appears to be dose-dependent, to generate permanent damage at high doses and minor damage at intermediate doses, to have possibly stimulatory effects at low doses (Arena et al., 2014a). For convenience, IR are calculated as an average (i.e., absorbed dose), nevertheless, irradiation can affect different areas of DNA, making difficult to draw a general pattern and to predict the resulting response (Kumagai et al., 2000; Maity et al., 2005; Kovalchuk et al., 2007). In order to investigate plant responses to IR, several experiments have been performed using Earth ground-based facilities mainly on seeds and seedlings (Kim et al., 2004; Zaka et al., 2004; Maity et al., 2005; Kim et al., 2011; El-mahrouk and El-sheikh, 2013; Marcu et al., 2013a; Marcu et al., 2013b; Fan et al., 2014; Van Hoeck et al., 2017). These studies showed that IR affects plant growth, development and reproduction, influencing anatomy, morphology and physiology (Kovalchuk et al., 2000; Yu et al., 2007; Real, 2018). Components of the photosynthetic apparatus such as electron transport carriers, light-harvesting complexes, carbon reduction cycle enzymes, chlorophyll a and b, were demonstrated to be sensitive to IR in *Capsicum annuum* and *Lactuca sativa* (Kim et al., 2004; Marcu et al., 2013a). Specifically, Kim et al., 2004 found that 2, 4 and 8 Gy of gamma rays enhanced the development of red pepper seedlings (germination, stem length and diameter, leaf area) and altered the compositions of photosynthetic pigments (chlorophylls and carotenoids) as well as the activities of antioxidant enzymes (superoxide dismutase and glutathione reductase). In addition, also Marcu et al., 2013a found that exposing lettuce seeds at doses ranging from 2 to 30 Gy enhanced the photosynthetic pigments (chlorophyll a, chlorophyll b, carotenoids).

Although the effects of cosmic radiation on plant development and reproduction are reported in literature, such experiments refer mainly to seeds and seedlings as developmental stage chosen for irradiation. Radiation studies on developed plants are very few.

The design of plant genotypes able to cope with non-terrestrial conditions is desirable to overcome possible hindrance to growth of natural crops in space environments. Plants capable to deal with non-terrestrial conditions may be obtained by manipulation of regulators of specialized metabolite pathways like MYB transcription factors, known for their contribution to the increased complexity of land plants along evolution and recognized as key players in the modulation of flavonoids biosynthesis in response to all kind of stimuli. Anthocyanins, flavonoids deriving from the phenylpropanoid pathway, are water soluble vacuolar pigments present in different tissues of higher plants. In vegetative tissues they act as protective compounds in response to environmental stimuli (i.e. UV light, cold stress) and biotic stress, with an essential role in modulating the ROS-signaling pathways, that makes them also natural antioxidant, anti-inflammatory and health-promoting biomolecules (Brunetti et al., 2013).

Tomato (*Solanum lycopersicum* L.) is one of the most cultivated vegetables worldwide and its fruits are the largest dietary source of lycopene, ascorbic acid, alpha-lipoic acid, choline, folic acid and lutein (Bognar et al., 2013; Mirdehghan and Valero, 2017). Accumulation of flavonoids in tomato fruits is sub-optimal and, with some exception, anthocyanins are not present (Jones et al., 2003; Mes et al., 2008). For these reasons, over the last decades, several attempts to improve anthocyanins content in tomato have been carried on, reporting anthocyanin accumulation in fruits and others organs, by both biotechnological approaches and conventional breeding (Bovy et al., 2002; Mathews et al., 2003; Butelli et al., 2008; Mes et al., 2008; Povero et al., 2011; Schreiber et al., 2012; Kiferle et al., 2015; Su et al., 2016; Blando et al., 2019; Jian et al., 2019; Colanero et al., 2020). There is growing interest in the design of food crops with improved levels and composition of these anti-oxidant nutraceuticals for space agriculture applications, as well (Gómez et al., 2021).

In the present work, we characterized the phenotypic effects of IR through administration of gamma radiation on the engineered *Solanum lycopersicum* determinate miniature-dwarf cultivar ‘MicroTom’ expressing a constitutive single copy of the *PhAN4* MYB gene from *Petunia hybrida* (Pagliarello et al., 2023). Thanks to dwarf determined growth (i.e., approximately 15 cm height at maturity) and short life cycle (90-100 days from sowing to fruit ripening), MicroTom is ideal in view of both ground-based and space investigations. These engineered plants have demonstrated bioaccumulation of anthocyanins in plant tissues inducing higher antioxidant capacity of ripe fruits (Pagliarello et al., 2023). Wild type and the engineered AN4-M and AN4-P_2_ MicroTom lines have both common and differential traits. In brief, life-cycle traits, such as the duration of vegetative state (i.e., time from sowing to flower development) and time from sowing to fruit development do not differ significantly among the three genotypes. On the contrary, vegetative traits, such as height, number of leaves and leaf area are significantly different, with AN4-M and, more markedly, AN4-P_2_ plants being characterized by a smaller habitus than wild type. The maximum fluorescent yield (Y_Fm) is in the range 0.65 - 0.85, that is described as correlated to healthy leaves, for all the three genotypes (Plant Stress Kit User’s Guide, Opti-sciences, Hudson, USA). Yield traits (i.e., number of flowers, number of fruits, diameter and weight of fruits) are not statistically different among the three genotypes, with the exception of the production of seeds, that is significantly higher in AN4-M plants compared to both wild type and AN4-P_2_.

A model of *PhAN4* engineered MicroTom cells (i.e., hairy root cultures) has already demonstrated a substantial tolerance to static magnetic field (SMF) and to irradiation with X and gamma rays (Villani et al., 2017; Desiderio et al., 2019) by a proteomic approach. Transcriptomic analysis indicated the regulation of traits related to resistance to oxidative and other abiotic stresses by *PhAN4* (Massa et al., 2022).

In this work, seeds and plants at the reproductive transition stage (i.e., 30 days after sowing, 30 DAS) from wild type and engineered MicroTom lines were exposed to IR doses simulated through the administration of gamma radiation (0 Gy, 0.5 Gy, 5 Gy or 30 Gy) generated by a ^60^Co source. Plant responses to gamma rays were evaluated following two non-destructive approaches: biometric parameters were monitored to detect changes occurred at both phenotypic and phenological level and multiparametric fluorescence-based indices were used to indirectly measure changes occurred at the primary and secondary metabolism level. Results give an indication that the manipulation of metabolic pathways can be an approach for adaptation of plants to extreme conditions, and to possibly obtain varieties for human consumption in outer space contexts.

## 2 Materials and methods

### 2.1 Plant material and growth conditions

*Solanum lycopersicum* miniature-dwarf-determinate cultivar ‘MicroTom’ (Scott and Harbaugh, 1989; Botella and Botella, 2016) engineered to accumulate anthocyanins by the constitutive expression of the R2R3-MYB gene *AN4* from *Petunia hybrida* (*PhAN4*; GenBank: HQ428105.1) was used. The third (T_3_) generation progeny of two primary transformants harboring one copy of *PhAN4*, namely, AN4-M (M: magenta color of fruits at the breaker + 7 days stage; homozygous) and AN4-P_2_ (P: purple color of fruits at the breaker + 7 days stage; hemizygous) at the 30 DAS life stage and the corresponding seeds were evaluated. The engineered plants, display a purple pigmentation throughout their structure, from leaves and leaf veins, to stem and roots, to anthers and ovary at different extent depending on genotype, with AN4-M homozygous plants showing milder purple pigmentation and accumulation of anthocyanins compared to hemizygous AN4-P_2_ plants (Pagliarello et al., 2023). Wild type MicroTom was used as a control.

Plants were cultivated in a containment greenhouse biosafety level 2 (BSL2) at the ENEA Casaccia Research Center (Rome, Italy) in hydroponic conditions. Plants grew in mesh pots containing a mix of horticultural perlite and vermiculite (1:1). The hydroponic module consisted of a tank containing distilled water mixed with the nutrient solution and a submersible pump connected to a system of nozzles able to nebulize the nutrient solution near the pots. The Idrofill base (1 g/L) (K-Adriatica, Loreo, Rovigo, Italy) (NPK 10–5.23, 8% CaO, 2% MgO; microelements: B 0.01%, Cu 0.02%, Fe 0.02%, Mn 0.01%, Mo 0.001%, Zn 0.003%) was used as nutrient solution. Irrigation was controlled by a Gronode unit (Opengrow LDA, Viseu, Portugal). The brain of the Grolab system, through the Powerbot unit (electronic device controller, Opengrow LDA, Viseu, Portugal), activates the submersible pumps in the tank. To provide the correct quantity of water and nutrients to the roots, a pre-programmed water delivery regime of 10 minutes every 2 hours was used. The pH was maintained between 5.5 and 6.5 and electrical conductivity set between 2.2 and 2.5 dSm^-1^ (Groline monitor, Hanna Instruments, Italy). Natural light was integrated with artificial light (LumiGrow PRO 650 lamps, blue LED 70 µmol m^-2^ s^-1^, white LED 70 µmol m^-2^ s^-1^, red LED 160 µmol m^-2^ s^-1^) (LumiGrow, California, USA). The temperature was set as a function of the photoperiod: 16 hours of light at 25°C and 8 hours of dark at 16°C. All experimental trials were conducted in an environment with relative humidity control set at 60%.

### 2.2 Gamma irradiation

Irradiation tests were performed on dry seeds and on fully developed plants at the 30 DAS stage in a pool-type irradiation plant equipped with a ^60^Co gamma source in a high volume (7 m x 6 m x 3.9 m) shielded cell (Calliope irradiation facility, Fusion and Nuclear Safety Technologies Department, ENEA, Rome, Italy). The source emits radiations consisting of two gamma photons with a mean energy of 1.25 MeV (Baccaro et al., 2019). Fricke dosimetric system was employed for the determination of the absorbed dose and of the dose rate during the irradiation tests. Samples (20 seeds/genotype/dose) and plants (5 plants/genotype/dose) were acutely irradiated at room temperature with either 0.5 Gy (dose rate: 6 Gy/h), 5 or 30 Gy (dose rate: 60 Gy/h). Due to the complexity in predicting the precise dose to which plants may be subjected in outer space missions, we hypothesized different scenarios of cultivation trials. In these scenarios (**Table 1**), we considered the most recent value obtained from RAD on board on the Curiosity Rover at Mars Gale crater for GCRs (0.210 ± 0.040 mGy/day) and the large event occurred in October 1989 for SEPs (1,454 mGy/hour) (Hassler et al., 2014; Chancellor et al., 2018). With the only exception of irradiation, non-irradiated and irradiated seeds and plants went through the same procedures (i.e., including the transfer to the irradiation facility), to exclude differential environmental effects. After irradiation, both controls and treated plants were returned to the greenhouse. Seeds were placed into *Petri* dishes on filter paper and soaked in the dark at 20°C to induce germination. The percentage of plantlets (PP) germinated at 15 days after sowing (DAS) was calculated as the first evaluation of irradiation effects. Each sample was compared to the non-treated controls of the same genotype to assess statistically significant radiation effects.

**Table 1:** Scenario hypothesized for cultivation trials on Mars surface.

Scenario 1		
**Seeds**	A ground-based or cultivation trial conducted in a completely shielded area	0 Gy
	Seeds stored for 1 year in a shielded area	0.5 Gy
	Seeds sowed in a greenhouse on Mars surface to benefits of solar PAR (GCRs + 1 SEP of 3 hours)	5 Gy
	Seeds sowed in a greenhouse on Mars surface to benefits of solar PAR (GCRs + 1 SEP of 2 days)	30 Gy
Scenario 2		
**Plants at 30 DAS**	A ground-based or cultivation trial conducted in a completely shielded area	0 Gy
	Plants sowed in a completely shielded area and transplanted at 30 DAS in a greenhouse on Mars surface to benefits of solar PAR (GCRs + 1 SEP of 15 mins)	0.5 Gy
	Plants sowed in a completely shielded area and transplanted at 30 DAS in a greenhouse on Mars surface to benefits of solar PAR (GCRs + 1 SEP of 3 hours)	5 Gy
	Plants sowed in a completely shielded area and transplanted at 30 DAS in a greenhouse on Mars surface to benefits of solar PAR (GCRs + 1 SEP of 2 days)	30 Gy

### 2.3 Non-destructive morphometric analyses

Plant response was analyzed through non-destructive morphometric analyses. Each plant was characterized by the following parameters: plant height (measured using a digital calliper), overall leaf area (measured using Image J version 1.8.0, National Institute of Health, USA), number of leaves, number of flowers, and number of fruits, fruit weight at the ripe stage (5 fruits per plant), equatorial diameter of fruits (5 fruits per plant, determined by a digital calliper), number of seeds (from 5 fruits per plant).

In the case of analyses on offspring of irradiated seeds, morphometric evaluations initiated 30 days after sowing (DAS) and were carried on weekly until fruit set. On the contrary, in the case of analyses on irradiated plants, morphometric evaluations initiated right after irradiation (i.e., after 10 minutes, 1 hour, 3 hours) and continued weekly until fruits set (DAI: Days After Irradiation). The fruit-related parameters were collected at the red ripe stage.

### 2.4 Non-destructive fluorimetric analyses

To investigate how irradiation may influence metabolic processes, non-destructive fluorimetric measurements were conducted using the Multiplex Research™ device (Force-A, Orsay, France). The following indices were monitored with a whole-plant approach: SFR, ANTH and NBI (Ghozlen et al., 2010; Tremblay et al., 2012). The SFR index is linked to the chlorophyll (Chl) concentration of leaves and increases with increasing Chl concentration. The ANTH index is proportional to the anthocyanin content. The NBI index represents the ratio between the primary and secondary metabolism (Chl index/FLAV index, the latter being proportional to flavonols). In the case of analysis on offspring of irradiated seeds, evaluations initiated 30 days after sowing (DAS) and were carried on weekly until fruit set. In the case of irradiated plants, analysis started right before and right after irradiation (i.e., after 10 minutes, 3 hours, 24 hours) and continued weekly until fruits set (DAI: Days After Irradiation).

### 2.5 Non-destructive fluorescent emission measurements

To track the performances of the photosynthetic apparatus and its capacity to recover from the radiative stress, the maximum fluorescence yield (Y_Fm) was monitored using the Plant Stress Kit (PSK) (Opti-sciences, Hudson, USA). The device detects the fluorescence emission through a sensor applied onto fully developed leaves. Since the suppression of photosynthesis is triggered in the context of the short-term reversible non-specific stress response to IR mounted by the plant from the first minutes to a few days after irradiation (Gudkov et al., 2019), analysis started right before and continued right after irradiation (i.e., after 10 minutes, 3 hours, 24 hours) until 7 DAI. This investigation was inapplicable in the case of offspring of irradiated seeds.

### 2.6 Data analysis

To evaluate the effects of the treatments, at each time point, *intra*-genotype analysis was performed and data related to plant height, overall leaf area, number of leaves and number of flowers, were compared with the same-genotype untreated controls, by two-way ANOVA analysis of variance with multiple comparisons. Variance of number, weight, diameter of fruits and number of seeds, that had been recorded only once at the red ripe stage of fruits, were coherently analyzed by one-way Anova comparing for the same-genotype controls. *Inter*-genotype comparison was performed to make evaluations on the differential performances of the three genotypes in terms of the fruit-related parameters and were analyzed by one-way ANOVA. The Tukey’s *post-hoc* test was used as a statistical hypothesis testing during the ANOVA analyses. Significant differences were shown as asterisks, where *p-value ≤ 0.05, **p-value ≤ 0.01 and ***p-value ≤ 0.001 were indicated on respective graphs. GraphPad Prism version 8 (GraphPad Software, San Diego, CA, USA) was used for all graphical and statistical data processing.

## 3 Results

### 3.1 Evaluation of germination on offspring of irradiated MicroTom seeds

At 15 DAS, more than 70% of irradiated seeds had produced a plantlet, irrespective of genotype and irradiation dose (**Table 2**). Compared to non-irradiated same-genotype controls, PP did not change for both wild type and AN4-M after 30 Gy, and increased for wild type after 0.5 Gy (by 5%), and for AN4-P_2_ after 0.5 Gy and 30 Gy (by 2.5% and 5%, respectively). Exposure of seeds to 5 Gy caused a slight decrease of PP in all the genotypes (wild type: 5%; AN4-M: 5%; AN4-P_2_: 2.5%). Gemination decreased in AN4-M after 0.5 Gy, as well (10%).

**Table 2:** Plantlets percentage at 15 DAS.

0 Gy		0.5 Gy	5 Gy	30 Gy
**Wild type**	75%	80%	70%	75%
**AN4-M**	80%	70%	75%	80%
**AN4-P_2_**	72.5%	75%	70%	77.5%

### 3.2 Morphometric evaluation on offspring of irradiated MicroTom seeds

**Figure 1:**
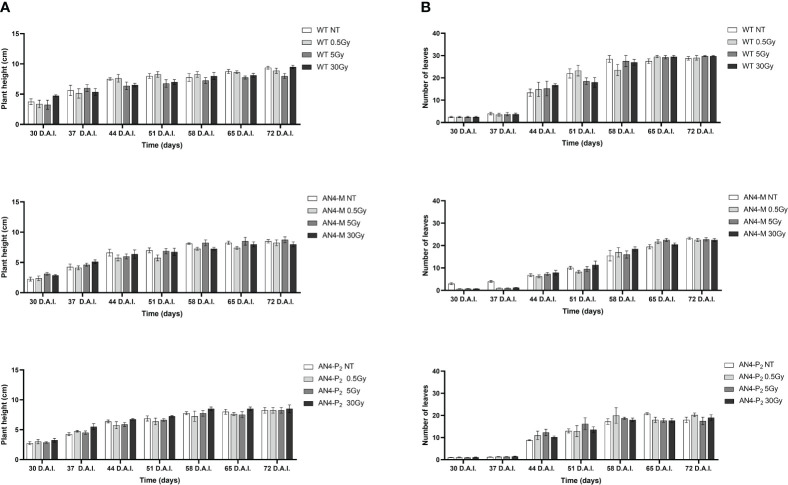
*Intra*-genotype analysis of plant height **(A)** and number of leaves **(B)** of wild type, AN4-M and AN4-P_2_ MicroTom along the observation period upon different irradiation treatments supplied to seeds. Mean values ± SE are shown ( n = 5). Analysis of variance was conducted by two-way ANOVA. Tukey’s *post-hoc* test was used as a statistical hypothesis testing. Asterisks indicate the statistically significant differences (Statistical relevance: *p<0.05; **p<0.001; ***p<0.0001).

**Figure 2:**
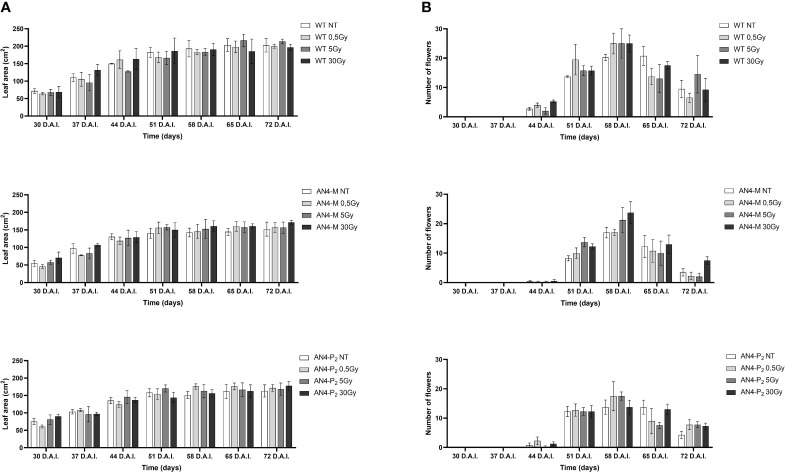
Intra-genotype analysis of leaf area **(A)** and number of flowers **(B)** of wild type, AN4-M and AN4-P2 MicroTom along the observation period upon different irradiation treatments supplied to seeds. Mean values ± SE are shown (n = 5). Analysis of variance was conducted by two-way ANOVA. Tukey’s post-hoc test was used as a statistical hypothesis testing. Asterisks indicate the statistically significant differences (Statistical relevance: *p<0.05; **p<0.001; ***p<0.0001).

Results obtained from offspring of irradiated MicroTom seeds are summarized in **Supplementary Table 1**. Throughout life cycle, offspring of irradiated seeds did not reveal statistically relevant differences for any of the morphometric parameters considered (i.e., height, number of leaves, leaf area, and number of flowers), independently of the administered adsorbed dose, compared with the respective same-genotype controls (**Figures 1**, **2**).

**Figure 3:**
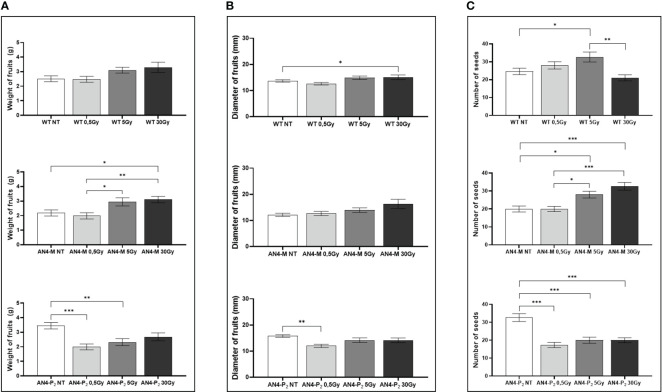
*Intra*-genotype analysis of weight **(A)**, diameter of fruits **(B)** and number of seeds **(C)** of wild type, AN4-M and AN4-P_2_ MicroTom ripe fruits upon the different irradiation treatments supplied to seeds. Mean values ± SE are shown ( n = 5). Analysis of variance was conducted by one-way ANOVA. Tukey’s *post-hoc* test was used as a statistical hypothesis testing. Asterisks indicate the statistically significant differences (Statistical relevance: *p<0.05; **p<0.001; ***p<0.0001).

At maturity, only few fruit-related parameters resulted to be affected, even in relation to the 30 Gy dose. The weight or diameter of fruits and seed yield resulted to be improved by irradiation in both AN4-M and wild type plants, especially after 5 and 30 Gy (**Figure 3**). On the contrary, a negative regulation of all the above-mentioned fruit-related traits was observed in offspring of AN4-P_2_, independently of the irradiation dose.

**Figure 4:**
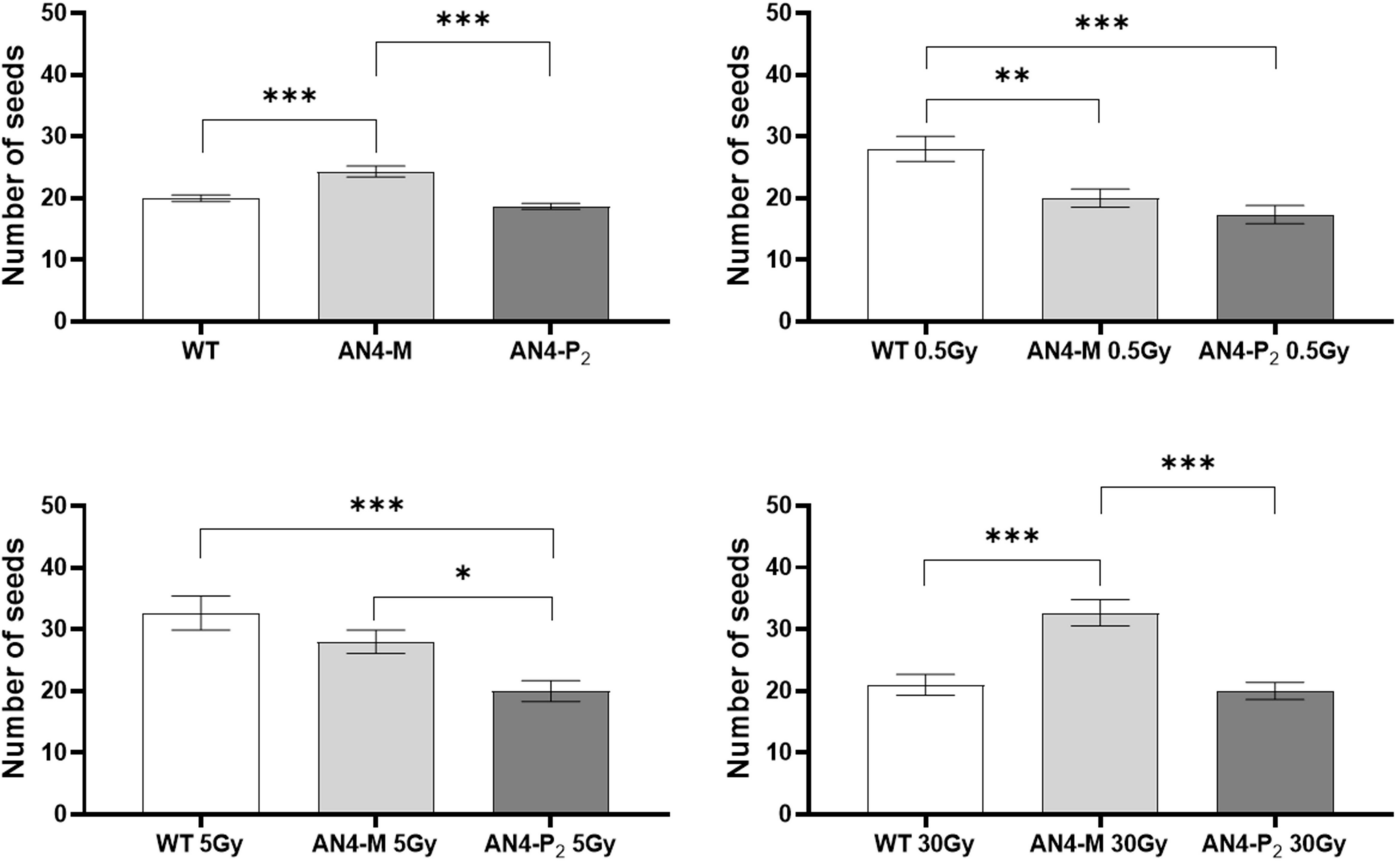
*Inter*-genotype analysis conducted on number of seeds of wild type, AN4-M and AN4-P_2_ MicroTom ripe fruits upon the different irradiation treatments supplied to seeds. Mean values ± SE are shown ( n = 5). Analysis of variance was conducted by one-way ANOVA. Tukey’s *post-hoc* test was used as a statistical hypothesis testing. Asterisks indicate the statistically significant differences (Statistical relevance: *p<0.05; **p<0.001; ***p<0.0001).

No statistical *inter*-genotype variation was observed for yield traits such as number of fruits, weight of fruits and diameter of fruits in irradiated *vs* controls for none of the genotypes, independently of the dose supplied (data not shown). The only yield trait to statistically vary among genotypes after irradiation was the number of seeds (**Figure 4**). After 0.5 Gy, the number of seeds produced by offspring of irradiated seeds resulted to be statistically higher in wild type than in both engineered lines, while, after 5 Gy, wild type was statistically superior only to the AN4-P_2_ genotype. After 30 Gy, the number of seeds produced by the AN4-M offspring was statistically higher than both wild type and AN4-P_2_, the latter resulting to be not statistically different between each other.

### 3.3 Fluorimetric evaluation of SFR, NBI and ANTH indices along life cycle of offspring of irradiated MicroTom seeds

The SFR and NBI indices progressively increased from 30 to 65 DAI (corresponding to 30 and 65 DAS, respectively) for all the genotypes despite irradiation, coherently with the undisturbed growth that had been visually observed (**Figures 1**, **2**).

**Figure 5:**
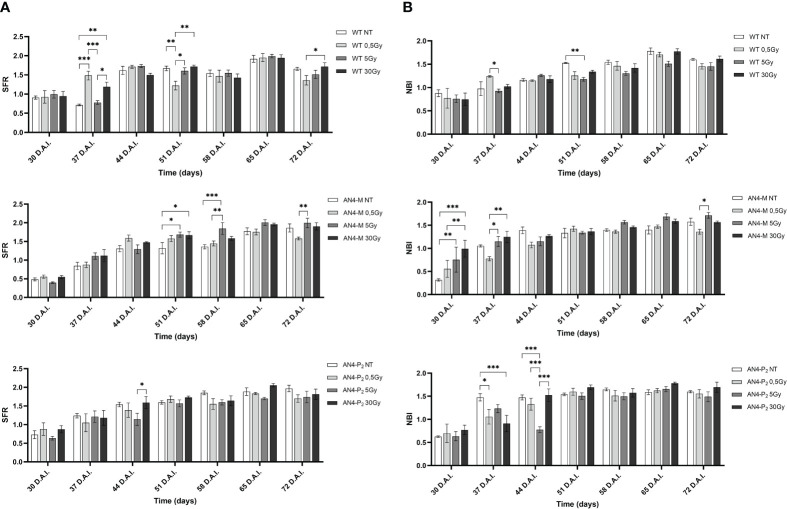
*Intra*-genotype analysis of the chlorophyll index (SFR) **(A)** and Nitrogen Balance index (NBI) **(B)** of wild type, AN4-M and AN4-P_2_ MicroTom along the observation period upon different irradiation treatments supplied to seeds. Mean values ± SE are shown ( n = 5). Analysis of variance was conducted by two-way ANOVA. Tukey’s *post-hoc* test was used as a statistical hypothesis testing. Asterisks indicate the statistically significant differences (Statistical relevance: *p<0.05; **p<0.001; ***p<0.0001).

The SFR sigmoidal-like trend with weekly periodicity of irradiated samples resulted similar to that of the same-genotype untreated controls (**Figure 5A**). In particular, an increase of SFR after 0.5 and 30 Gy was recorded at 37 DAI in wild type offspring, while a decrease was observed only after 0.5 Gy, at 51 DAI (51 DAS). An increase after the 5 and 30 Gy doses was recorded for AN4-M offspring at 51-58 DAI (51-58 DAS). In AN4-P_2_, no statistical differences with untreated same-genotype samples were recorded.

Variations of NBI values compared to untreated same-genotype controls occurred independently of genotypes (**Figure 5B**), with some differences. In particular, an increase was recorded after the 5 Gy dose for wild type at 51 DAI (51 DAS). AN4-M offspring experienced an increase of the NBI increase after 5 and 30 Gy at 30 DAI (30 DAS). In AN4-P_2_, a significant decrease of the NBI values was found at 37 DAI (37 DAS) after 0.5 and 30 Gy, while at 44 DAI (44 DAS) the 5 Gy dose caused a significant decrease of the index.

**Figure 6:**
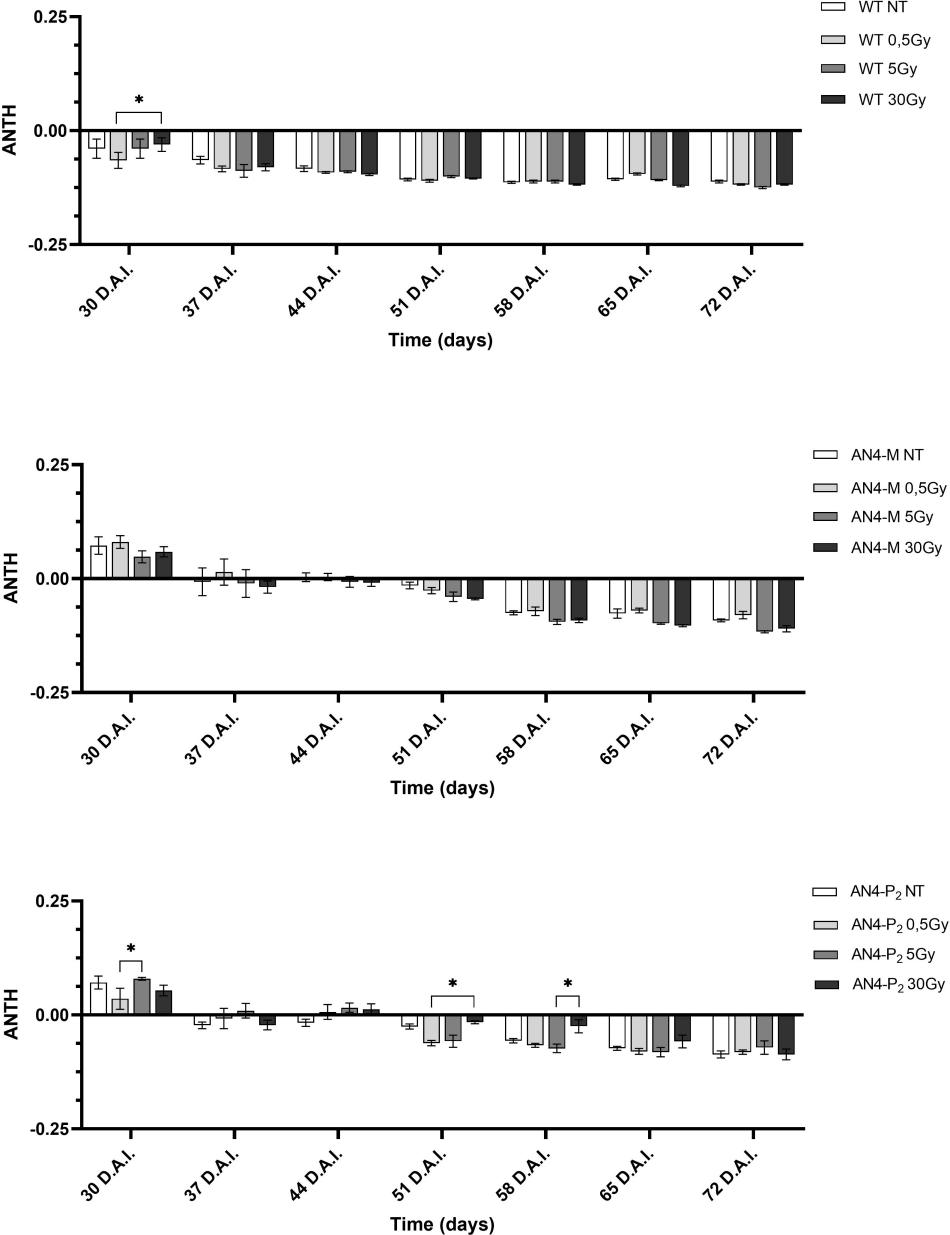
Intra-genotype analysis of the anthocyanin index (ANTH) of wild type, AN4-M and AN4-P2 MicroTom along the observation period upon different irradiation treatments supplied to seeds. Mean values ± SE are shown (n = 5). Analysis of variance was conducted by two-way ANOVA. Tukey’s posthoc test was used as a statistical hypothesis testing. Asterisks indicate the statistically significant differences (Statistical relevance: *p<0.05).

As expected, due to the virtual absence of anthocyanins in wild type MicroTom, the ANTH index produced different curves for the wild type compared to the two transgenic genotypes along life cycle (**Figure 6**). ANTH values were constantly negative in wild type offspring, indicating the absence of anthocyanins, with no statistical relevance between irradiated and non-irradiated seeds independently of dose. AN4-M and AN4-P_2_ offspring of both irradiated and non-irradiated seeds were characterized by a sigmoidal curve with an inversion to negative values around 37-44 DAI (37-44 DAS). No statistical relevance was observed in offspring of neither AN4-M or of AN4-P_2_ offspring compared to same-genotype controls. Slight statistical differences were found only for AN4-P_2_ offspring and only among irradiated samples (i.e., between 0.5 and 5 Gy at 30 DAI; between 0.5 and 30 Gy at 51 DAI; between 5 and 30 Gy at 58 DAI).

The evaluation of all the fluorimetric parameters (SFR, ANTH and NBI) at maturity showed that the irradiation of seeds did not produce significant variations in offspring compared to untreated same-genotype controls irrespective of genotype and dose (**Supplementary Table 1**).

### 3.4 Morphometric evaluation of 30 DAS irradiated MicroTom plants

Independently from genotype, plants survived the immediate consequences of ionizing radiation and were able to flower and complete their own life cycle. Results obtained from plants irradiated at 30 DAS are summarized in **Supplementary Table 2**.

**Figure 7:**
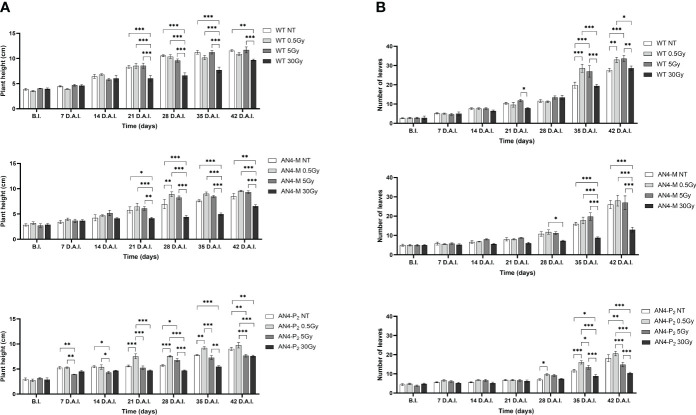
*Intra*-genotype analysis of plant height **(A)** and number of leaves **(B)** of wild type, AN4-M and AN4-P_2_ MicroTom along the observation period upon different irradiation treatments supplied to plants at 30 DAS. Mean values ± SE are shown ( n = 5). Analysis of variance was conducted by two-way ANOVA. Tukey’s *post-hoc* test was used as a statistical hypothesis testing. Asterisks indicate the statistically significant differences (Statistical relevance: *p<0.05; **p<0.001; ***p<0.0001).

Analysing plant height along life cycle, except for plants receiving 30 Gy, no significant variations were found in wild type, compared to same-genotype untreated controls, independently of the absorbed dose. In AN4-M plants, significant increases of plant height were observed only at 28 DAI (58 DAS) after the 0.5 Gy dose, compared to same-genotype untreated controls. In AN4-P_2_ plants, the 0.5 Gy dose determined significant increases of height compared to both untreated same-genotype controls starting from 21 until 35 DAI (51-65 DAS), while the 5 and 30 Gy doses determined a decrease of height compared to same-genotype controls starting from 7 DAI until 21 DAI (37-51 DAS) and 35 DAI until 42 DAI (65-72 DAS), respectively (**Figure 7A**).

Analysing variation of number of leaves throughout life cycle, no significant differences were found until 35 DAI (65 DAS), irrespective of genotype. From this point on, differential variations were found depending on genotype. Significant increases of the number of leaves were observed for wild type plants after 0.5 and 5 Gy between 35 and 42 DAI (65-72 DAS), compared to the same-genotype controls. In AN4-M, variation of the number of leaves was observed only after 30 Gy at 35 and 42 DAI. In AN4-P_2_ plants, we observed an increase of number of leaves after 0.5 Gy at 35 DAI and a decrease of the same parameter after the irradiation with 5 and 30 Gy at 35 and 42 DAI, respectively (**Figure 7B**).

**Figure 8:**
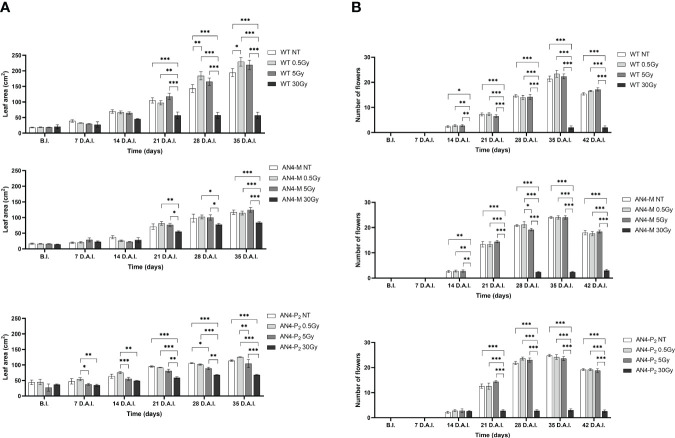
*Intra*-genotype analysis of leaf area **(A)** and number of flowers **(B)** of wild type, AN4-M and AN4-P_2_ MicroTom along the observation period upon different irradiation treatments supplied to plants at 30 DAS. Mean values ± SE are shown ( n = 5). Analysis of variance was conducted by two-way ANOVA. Tukey’s *post-hoc* test was used as a statistical hypothesis testing. Asterisks indicate the statistically significant differences (Statistical relevance: *p<0.05; **p<0.001; ***p<0.0001).

Except for the 30 Gy dose, that determined a decrease of leaf area in all the genotypes, only the 0.5 Gy dose determined an effect in wild type compared to same-genotype untreated controls (significant increase of leaf area from 28 DAI to 35 DAI; 58-65 DAS). No significant variations were recorded in AN4-M plants. Slightly significant decreases of this parameter were recorded for the AN4-P_2_ after the 5 Gy dose compared to same-genotype untreated controls 28 DAI (**Figure 8A**).

**Figure 9:**
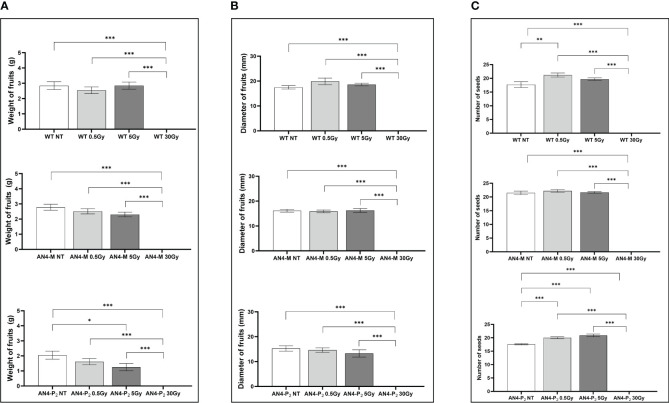
*Intra*-genotype analysis of weight **(A)**, diameter of fruits **(B)** and number of seeds **(C)** of wild type, AN4-M and AN4-P_2_ MicroTom ripe fruits upon the different irradiation treatments supplied to plants at 30 DAS. Mean values ± SE are shown ( n = 5). Analysis of variance was conducted by one-way ANOVA. Tukey’s *post-hoc* test was used as a statistical hypothesis testing. Asterisks indicate the statistically significant differences (Statistical relevance: *p<0.05; **p<0.001; ***p<0.0001).

In relation to the number of flowers (**Figure 8B**) and number and diameter of fruits, except for the 30 Gy dose that determined a significant decrease of these parameters in all the genotypes, no statistical variations were observed compared to untreated controls. At maturity, the 30 Gy dose determined an overall decrease of values related to both vegetative and yield traits, independently of genotype (**Supplementary Table 2**, **Figure 9**). The 30 Gy dose prevented plants from completing a seed-to-seed cycle, otherwise fulfilled, due to poor flowering and no fruit production. Compared to their same-genotype untreated controls, wild type plants showed significant increase in number of leaves after the 0.5 and 5 Gy doses, leaf area and number of seeds after the 0.5 Gy doses. AN4-M plants resulted completely unaffected by both the 0.5 and 5 Gy doses. The number of seeds significantly increased in AN4-P_2_ plants after the 0.5 and 5 Gy doses (**Figure 9**).

**Figure 10:**
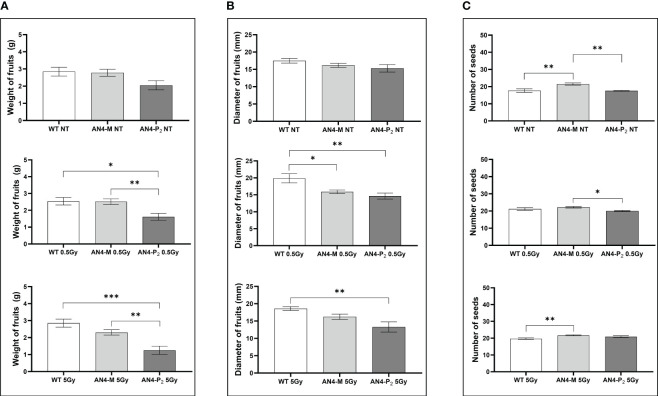
Inter-genotype analysis conducted on weight of fruits **(A)**, diameter of fruits **(B)** and number of seeds **(C)** of wild type, AN4-M and AN4-P2 MicroTom ripe fruits upon the different irradiation treatments supplied to plants at 30 DAS. Mean values ± SE are shown (n = 5). Analysis of variance was conducted by one-way ANOVA. Tukey’s post-hoc test was used as a statistical hypothesis testing. Asterisks indicate the statistically significant differences (Statistical relevance: : p<0.05; *: p<0.001; ***: p<0.0001).

Relevant statistical *inter*-genotype variations referred to three yield parameters: weight, diameter of fruits and number of seeds (**Figure 10**). After both the 0.5 and 5 Gy doses, the AN4-M fruits were not statistically different from wild type in terms of weight, while AN4-P_2_ fruit weight was statistically lower than both wild type and AN4-M. In terms of diameter, fruits of wild type showed statistically relevant higher values compared to both AN4-M and AN4-P_2_ fruits after the 0.5 Gy dose, while after the 5 Gy dose, the values for wild type are statistically higher only compared to AN4-P_2_. The number of seeds was positively influenced by both 0.5 and 5 Gy doses mainly in wild type and AN4-P_2_ plants. This led the AN4-M plants to maintain their standard statistical superiority only to the AN4-P_2_ plants after the 0.5 Gy, and to wild type after the 5 Gy dose treatment.

### 3.5 Fluorimetric evaluation of SFR, NBI and ANTH indices in irradiated MicroTom plants

**Figure 11:**
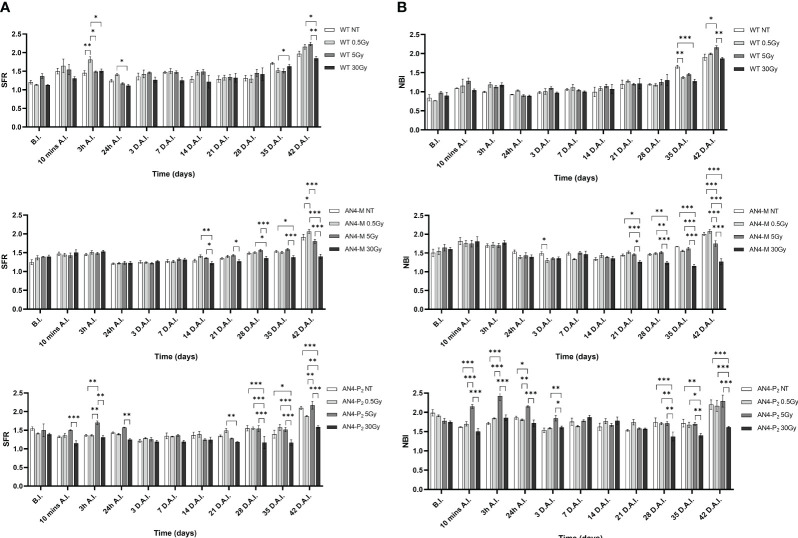
*Intra*-genotype analysis of the chlorophyll index (SFR) **(A)** and Nitrogen Balance index (NBI) **(B)** of wild type, AN4-M and AN4-P_2_ MicroTom along the observation period upon different irradiation treatments supplied to plants at 30 DAS. Mean values ± SE are shown ( n = 5). Analysis of variance was conducted by two-way ANOVA. Tukey’s *post-hoc* test was used as a statistical hypothesis testing. Asterisks indicate the statistically significant differences (Statistical relevance: *p<0.05; **p<0.001; ***p<0.0001).

The SFR and NBI value trend of irradiated plants along life cycle was similar to that of the same-genotype untreated controls independently of genotype (**Figure 11**). Regardless of the irradiation dose, the values of the SFR and NBI indices progressively increased until 3 h A.I. (After Irradiation) as a prosecution of the data retrieved before irradiation (B.I.), for all the three genotypes. Starting from 24 h A.I., a clear reset of the SFR values was recorded toward lower values, progressively increasing afterwards, until the end of the measurements (**Figure 11A**). Furthermore, 3 h A.I. wild type plants subjected to the 0.5 Gy dose and AN4-P_2_ plants subjected to the 5 Gy dose, showed an increase of the SFR index compared to same-genotype untreated controls, while no changes occurred in AN4-M plants. Thereafter, both AN4-M and AN4-P_2_ plants showed statistically significant changes only at the end of the measurements (i.e., from 35 DAI; 65 DAS).

Starting from 24 h A.I., a reset of the NBI values toward lower values was recorded, progressively increasing afterwards, until the end of the measurements for wild type and AN4-M plants (**Figure 11B**). AN4-P_2_ plants did not show the reset of NBI values after 24 h A.I. observed for the other genotypes. In addition, AN4-P_2_ plants that had received the 5 Gy dose, produced NBI values significantly overwhelming those recorded for the same-genotype controls and for the other doses especially in the interval 10 min-24 hours A.I. The 30 Gy dose had a significant negative effect on the NBI index of AN4-P_2_ plants starting from 28 DAI (58 DAS) until the end of the detection period. This negative effect was also found in AN4-M plants starting from 21 DAI (51 DAS). Conversely, at 35 DAI (65 DAS) both 0.5 and 30 Gy caused a decrease of the NBI index in wild type plants. However, from 42 DAI (72 DAS), the 5 Gy dose had an increasing effect on the NBI in wild type plants.

**Figure 12:**
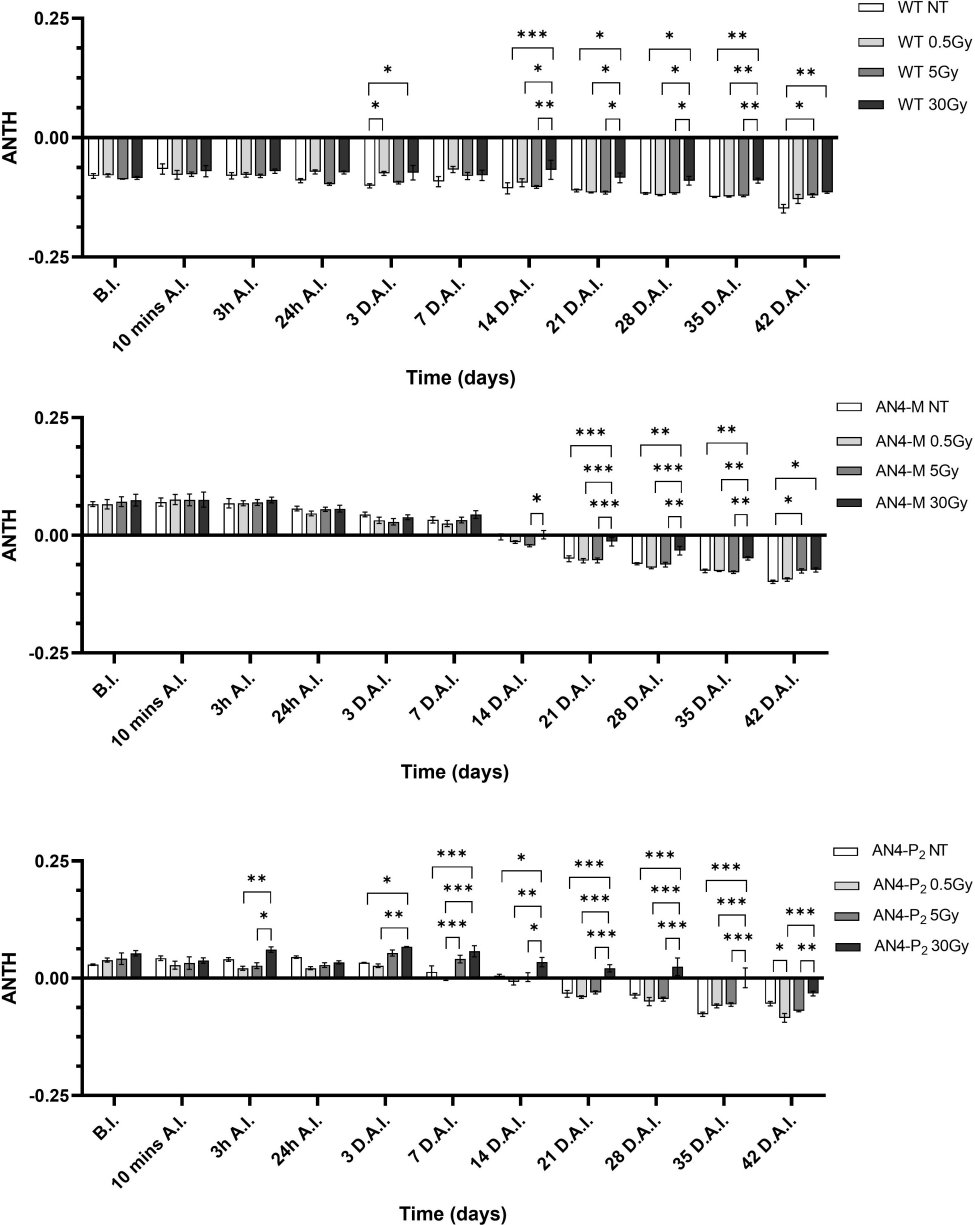
*Intra*-genotype analysis of the anthocyanin index (ANTH) of wild type, AN4-M and AN4-P_2_ MicroTom along the observation period upon different irradiation treatments supplied to plants at 30 DAS. Mean values ± SE are shown ( n = 5). Analysis of variance was conducted by two-way ANOVA. Tukey’s *post-hoc* test was used as a statistical hypothesis testing. Asterisks indicate the statistically significant differences (Statistical relevance: *p<0.05; **p<0.001; ***p<0.0001).

As expected, and as seen in the offspring of irradiated seeds, the ANTH index values defined different curves for the wild type compared to the two transgenic lines (**Figure 12**). Indeed, while ANTH values were constantly negative along life cycle in wild type, indicating absence of anthocyanins, engineered plants were characterized by a sigmoidal curve with a value inversion point between positive and negative ratios, 7 and 14 DAI (37 and 44 DAS, respectively), in correspondence of flowering. AN4-M plants showed increases of the ANTH index after the 30 Gy starting from 14 DAI (44 DAS). AN4-P_2_ plants, as well, experienced increases of the index after the 30 Gy doses, but starting from 3 DAI (33 DAS) until day 42 DAI (72 DAS).

At maturity, compared to same-genotype controls, wild type plants showed an increase of NBI (under the 5 Gy dose). In AN4-M plants, an increase of the SFR (after the 0.5 Gy dose) and the ANTH (after the 5 and 30 Gy doses) indices, and a decrease of the NBI index (both after the 5 and 30 Gy doses) were observed. In AN4-P_2_ plants, a decrease of all the three indices (i.e., ANTH index under the 0.5 Gy dose, SFR and NBI indices under the 30 Gy dose) was found (**Supplementary Table 2**).

### 3.6 Fluorimetric evaluation of photosynthetic performance in irradiated MicroTom plants

**Figure 13:**
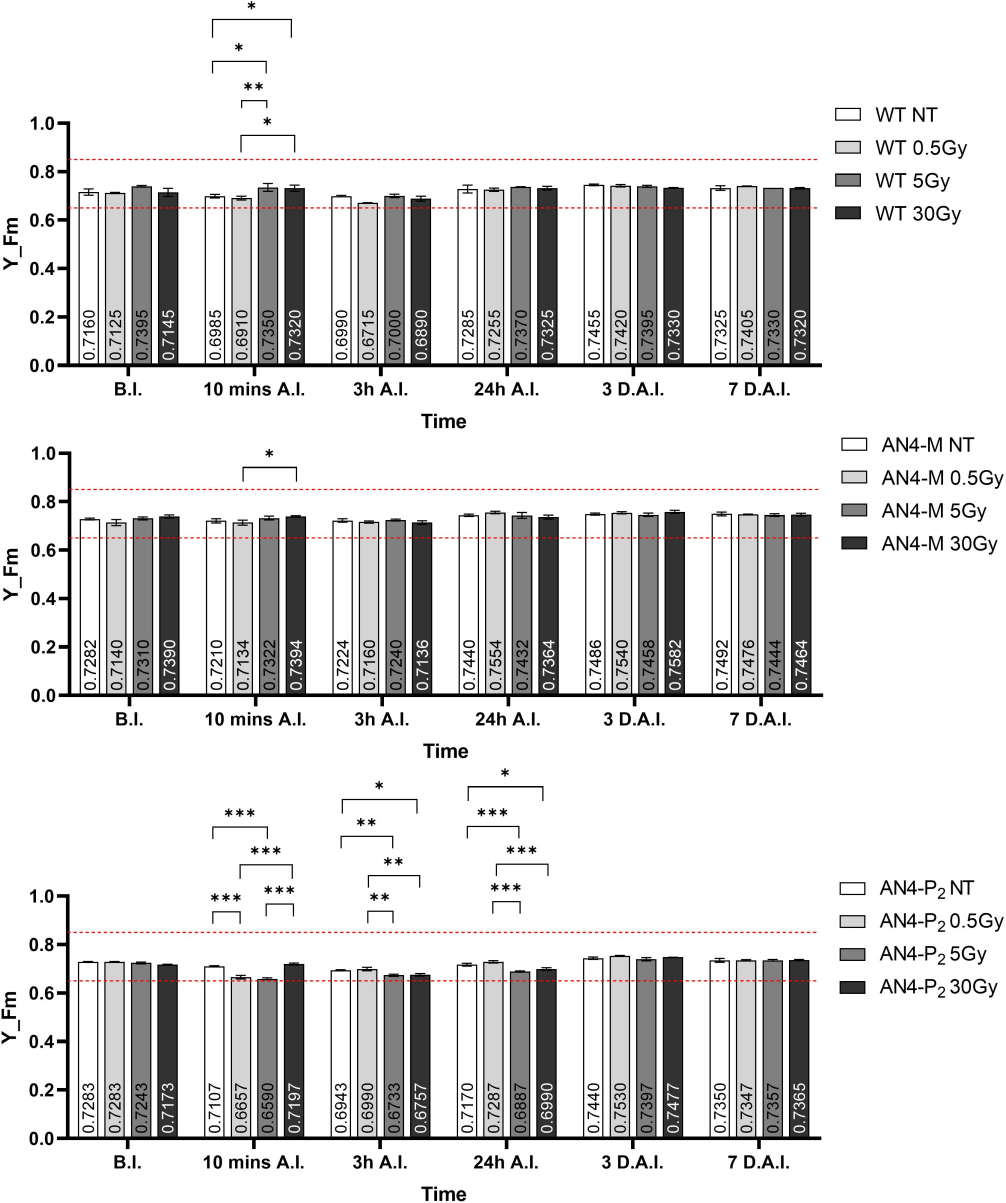
*Intra*-genotype analysis of the maximum fluorescence yield index (Y_Fm) of wild type, AN4-M and AN4-P_2_ MicroTom along the observation period upon different irradiation treatments supplied to plants at 30 DAS. Mean values ± SE are shown ( n = 5). Analysis of variance was conducted by two-way ANOVA. Tukey’s *post-hoc* test was used as a statistical hypothesis testing. Asterisks indicate the statistically significant differences (Statistical relevance: *: p<0.05; **: p<0.001; ***: p<0.0001).

Despite the NBI and SFR fluorimetric indices underwent some variations in dependence of the genotype and treatment, the maximum fluorescent yield (Y_Fm) values remained in the range described for healthy leaves, independently of genotype and radiation indicating no stress induction by irradiation (**Figure 13**). Statistically relevant variations of Y_Fm compared to the same-genotype controls were detected within 24 hours. Wild type underwent an increase of the Y_Fm index after the 5 and 30 Gy doses 10 minutes after irradiation, while irradiated AN4-P_2_ plants showed a decrease of the index after the 0.5 and the 5 Gy doses and continued to experience a decrease 3 and 24 hours A.I. after both the 5 and the 30 Gy dose. On the other hand, the AN4-M plants showed no variations compared to the untreated same-genotype controls.

## 4 Discussion

In support of NASA’s goals for human exploration and sustained presence on the Moon and beyond, new research is needed to provide basis for freshly grown crop plants (Wolff et al., 2014; Massa et al., 2016; Imhof et al., 2018; Marzioli et al., 2020). The ideal space crop plants should primarily be able to cope with harmful IR, in order to complete life cycle and, subsequently, to provide value-added fresh food sustaining humans against IR-related oxidative stress. To these ends, efforts to figure out how genetic engineering would contribute to improve plant performances in an extra-terrestrial environment are needed.

Tomato (*Solanum lycopersicum* L.) is one of the most cultivated crops worldwide and the largest dietary source of lycopene, ascorbic acid, alpha-lipoic acid, choline, folic acid and lutein (Mirdehghan and Valero, 2017). Among plant secondary metabolites, anthocyanins show great interest in the design of food crops (among which tomato) with improved levels and composition of anti-oxidant nutraceuticals for space agriculture applications (Gómez et al., 2021). Notably, anthocyanins are described to help protecting plants themselves from several abiotic stresses such as IR (Kaur et al., 2023). Therefore, we have engineered the tomato dwarf determinate variety ‘MicroTom’, that is considered as one of the suitable varieties for space application of agriculture, to express PhAN4, a MYB-like transcription factor able to restore the biosynthesis of anthocyanins in tomato (Pagliarello et al., 2023).

Plant response to IR largely depends on the type of radiation, dose and dose rate (Caplin and Willey, 2018). Furthermore, species, cultivar, plant developmental stage, irradiated tissue or organ, ploidy level and the nature of IR-induced DNA damage may influence plant response and survival to IR (Kiong et al., 2008; De Micco et al., 2011). It has been reported that IR can influence the content of phenolic compounds (Arena et al., 2014b; De Micco et al., 2014b) and the activity of the photosynthetic apparatus (Kim et al., 2004; Marcu et al., 2013b). Clearly, although general considerations can be drawn, due to the involvement of different key factors, it is usually difficult to depict a concise and general plant response behavior to IR.

Precisely tracking variations of vegetative and yield traits of plants upon irradiation trials, together with the alteration of indices that are related to primary and secondary metabolism through a systematic method, is necessary to establish performance metrics. The systematic recording of morphometric data is indicated as the method to be followed in cultivation studies as it complies with the CRL (crop readiness level) proposed by Romeyn et al., 2019 to implement the “technology readiness level” (TRL) that is currently used by NASA and ESA in agriculture for space applications.

In our work, besides following the current guidelines, we have also recorded multiparametric fluorescence-based indices to indirectly measure changes occurring at the physiological level. The use of a multiparametric non-destructive approach is important to monitor the overall response of plants during a cultivation trial without imposing further damage and stress to plants in addition to IR. The measurement of the latter indices is advised in view of the validation of remotely controlled multiparametric fluorescence-based instruments in the future lunar outposts.

Cosmic IR simulated on a laboratory scale using ground-based facilities has been used, to date, to investigate responses of wild type MicroTom only at the seed stage through either X rays (i.e., low-LET radiation type, upon 0.3 - 100 Gy dose) or Ca ions (i.e., high-LET radiation type, 25 Gy dose) administration (De Micco et al., 2014b; Arena et al., 2019). Our experiments focused on investigating wild type and anthocyanin-accumulating MicroTom phenotypic response (i.e., vegetative and yield morphometric traits, primary and secondary metabolism indices) at both seed and plant (30 DAS, reproductive transition) stages. These experiments were accomplished through the administration of ^60^Co gamma radiation, that is characterized by higher energy speed and penetration power than other low-LET radiation such as X-rays (i.e., 2.5 MeV *vs* 82 keV), thus increasing the present knowledge in tomato responses to cosmic radiation components.

Our experiments confirmed that the seed represents a phenological stage that tolerates higher radiation doses than vegetative stages. Structure, low H_2_O content and high melatonin levels, have been mentioned as possible explanations for this resistance (Gudkov et al., 2016; Arena et al., 2019). Offspring of irradiated seeds showed significant variations of only yield traits, such as weight and diameter of fruits and number of seeds, being either improved (in wild type and AN4-M plants) or worsened (in AN4-P_2_), without impairing a normal fruit set and completion of seed-to-seed cycle upon any dose (**Supplementary Table 1**, **Figures 3**, **4**). These results show that absorbed doses up to 30 Gy with the features of the radiation administered in these experiments, do not induce structural aberrations of the seed able to prevent physiological functions of the offspring. Our results on irradiation of wild type seeds with a 30 Gy dose differ from those found by Arena et al., 2019, who found negative effects on germination percentage and plant height at 25 Gy. Although comparisons can be made, the characteristics of Ca ions and gamma rays perfectly explain the different response. Indeed, in Arena et al., 2019, isotope ^48^Ca-deriving radiation with an energy of 200 MeV/u and a dose rate of 1 Gy/min were used, while gamma rays from a ^60^Co source, with a mean energy of 1.25 MeV and a dose rate of 60 Gy/hour were used in this work. It is known that the radiation-induced plant behavior depends on radiation type, dose and dose-rate (Arena et al., 2014a). For this reason, also comparison with data from X rays administration (0.3 and 10 Gy, dose rate: 1 Gy/min) to wild type MicroTom seeds (De Micco et al., 2014b) is difficult. These authors reported that irradiation with 10 Gy significantly increased plant height, while both 0.3 and 10 Gy resulted in higher leaf area and number of leaves, while no recording of fruits parameters was considered in the study. In our experiments, significant variations have been found for wild type only in terms of seed production of offspring after the 5 Gy dose (**Supplementary Table 1**).

In the phenotypic response of offspring of irradiated seeds, the specific genetic asset turned out to be a factor influencing the response of MicroTom to IR. The AN4-M offspring either maintained or enhanced yield traits (i.e., improved weight and diameter of fruits upon 30 Gy and seed production upon 5 Gy) while maintaining smaller habitus than wild type, the ability to accumulate anthocyanins and to perform primary metabolism normally. Indeed, despite a late response was recorded through the SFR and NBI indices in offspring of irradiated wild type and AN4-M seeds (i.e., corresponding variations of the two indices upon either 0.5, or 5, or 30 Gy, at 51-58 DAI and 30-37 DAI, respectively; **Supplementary Table 1**, **Figure 4**, **5**), no significant variations of these and ANTH fluorimetric indices were recorded at maturity compared to the same-genotype control.

Chlorophyll level and oxidation, changes in chloroplasts number and size (all correlated to the calculation of the SFR and NBI indices) and proline mobilization, have been described to occur upon environmental and IR-related stress in plants (Kim et al., 2011, De Micco et al., 2014a, b; Dartnell et al., 2011; Kautz et al., 2014). Variations of the NBI index have been described as the consequence of the reallocation of nitrogen compounds from older to younger leaves in response to stress tolerance after irradiation and abiotic stress in general (Vijayakumari et al., 2016; Volkova et al., 2020). The adjustments of the SFR and NBI indices showed by wild type and AN4-M offsprings, sustained the photosynthetic performances within the typical range of healthy leaves along the whole life cycle (**Supplementary Figure 1**). No statistical differences of the ANTH index were observed in offspring of the engineered irradiated seeds along life cycle (**Figure 6**) compared to the same-genotype controls. Therefore, engineering of the anthocyanin pathway through the constitutive expression of the PhAN4 factor was able to sustain anthocyanin continuous accumulation without variations compared to same-genotype controls at any stage, possibly allowing a standardization of the crop harvest regardless of the absorbed dose.

To date, the physiological and biochemical processes that determine changes in growth and development of adult plants subjected to IR remain mostly unknown (Gudkov et al., 2019). In addition, research in the present field has been mainly performed on irradiated seeds. To fill the gap of knowledge about the phenotypic response of tomato plants to gamma rays, wild type and engineered plants at the reproductive transition stage (i.e., 30 DAS) were irradiated. This phenological stage coincides with flowering and is crucial for completing the entire life cycle and to study the effects of radiation on yield traits.

In plants, differently from offspring of irradiated seeds, the 30 Gy dose determined an overall depletion of phenotypic values related to both vegetative and yield traits at maturity, independently of genotype. Drastic decrease of the number of flowers and, consequently, of the number of fruits and fruit-related parameters were observed (**Supplementary Table 2**). These results show that an absorbed dose of 30 Gy with the features of the radiation administered in these experiments, heavily disturb physiological molecular functions of plants at this stage. Between 5 and 30 Gy, a limiting dose should exist that MicroTom in reproductive transition can’t handle in order to complete a seed-to-seed cycle. Due to logistical issues, it was not possible to examine the 5-30 Gy dose range in these experiments. In the future, it would be useful to investigate this point.

On the other hand, upon the 0.5 and the 5 Gy doses, plants were able to produce viable seeds (data not shown) and the second generation is currently being evaluated to increase the knowledge on the inherited effects of IR (manuscript in preparation). As in the case of offspring of irradiated seeds, the specific genetic asset turned out to be a factor influencing the response of MicroTom to IR. Indeed, along life cycle, early or late responses could be recorded through fluorimetric indices depending on genotype. If we exclude from the analysis the outcomes from the 30 Gy dose (i.e., a very unlikely condition, causing variations compared to non-irradiated controls in all the genotypes), differently from wild type and AN4-P_2_ plants, AN4-M responses could be statistically mostly limited to a very late response (42 DAI, corresponding to 72 DAS, when plants have fulfilled their seed-to-seed cycle) visible only upon the 5 Gy dose through a point decrease of the NBI index compared to non-irradiated controls. Wild type and AN4-P_2_ plants showed a statistically stronger and more complex response (i.e., early response 3 h AI, through a point increase of the SFR index upon 0.5 Gy, and late responses 3 DAI and 42 DAI through a decrease and an increase of the NBI index, respectively, in wild type upon 0.5 or 5 Gy; only early responses 3 DAI through a point variation of the SFR index and prolonged increases from 10 mins AI to 24 h AI of the NBI index upon 5 Gy in AN4-P_2_) (**Figures 11**, **12**).

Indeed, at maturity, the AN4-M plants did not show any variation for any trait compared to the same-genotype control. On the contrary, the wild type plants showed improvement in morphometric traits both upon the 0.5 and the 5 Gy (e.g., number of leaves, leaf area and number of seeds upon 0.5 Gy; number of leaves upon 5 Gy) with the number of leaves (i.e., the value of the trait affected upon both doses) increasing from 0.5 Gy to 5 Gy. The AN4-P_2_ showed progressive decrease of values and for more traits from 0.5 to 30 Gy (e.g., plant height, number of leaves, diameter of fruits) (**Supplementary Table 2**).

Weight and diameter of fruits are yield traits not statistically different among the three genotypes in standard conditions. *Inter*-genotype comparison demonstrated that upon the 0.5 and 5 Gy doses, wild type and AN4-M plants remained mostly not statistically different (differently from AN4-P_2_, where both values decreased). The production of seeds was not affected in AN4-M fruits (remaining significantly higher than wild type upon 5 Gy), while it was enhanced in AN4-P_2_ plants, with the 5 Gy dose leading to a higher seed production compared to wild type, and to no statistical difference compared to AN4-M plants (**Figure 10**). Therefore, the AN4-M and AN4-P_2_ plants, respectively, maintained or improved yield traits while retaining a smaller habitus than wild type, the ability to accumulate anthocyanins, and to perform primary metabolism normally (**Supplementary Table 2**; **Figures 11**–**13**). These features can be all considered beneficial for cultivation in the confined environments that will be dedicated to space agriculture. On the contrary, the response of wild type plants to 0.5 and 5 Gy doses through the increase of number of leaves or leaf area, respectively, would be detrimental in view of the necessary optimization in future space cultivation systems. The increase in seed production upon the 0.5 Gy dose has the only effect to make wild type not statistically different from AN4-M.

The only decrease of the ANTH index upon 0.5 Gy showed by AN4-P_2_ plants (p<0.05) may be associated with the oxidative bleaching and degradation of the polyphenolic structure of anthocyanins as the result of the condensation of ascorbic acid at carbon-4 (C4) (Farr and Giusti, 2018). We can hypothesize that AN4-M plants (that did not show any decrease of the ANTH values) are more protected from the oxidative bleaching of anthocyanins compared to AN4-P_2_ as a consequence of their lower content in anthocyanins and higher availability of other antioxidant flavonoids as their precursors (data not shown, manuscript in preparation), as explained by Farr and Giusti, 2018 (**Supplementary Figure 2**). Interesting to note that the 30 Gy dose, differently from seeds, is able to improve the anthocyanin content of both AN4-M and AN4-P_2_ compared to controls from 21 DAI and 3 DAI, respectively.

As happened for offspring of irradiated seeds, photosynthetic performances were not influenced by the irradiation of plants, independently of genotype (**Figure 13**).

It can be concluded that, despite MicroTom demonstrated to be a very inherently robust plant in terms of phenotypic response to gamma rays irradiation, the AN4-M seeds and plants, showed advantages over wild type in terms of future application in space agriculture: negligible variation of fluorimetric parameters related to primary metabolism with no alteration or improvement of yield traits at maturity while maintaining smaller habitus than wild type, biosynthesis of anthocyanins and maintained levels of these compounds compared to non-irradiated controls of the same age (**Supplementary Tables 1, 2**). These results highlight that the anthocyanin enrichment of MicroTom through engineering may be a suitable approach for space agriculture application. In particular, AN4-M seeds and plants are favorable in different radiation scenarios based on their maintenance of productivity/size ratio and on their ability to perform anthocyanin biosynthesis.

The principal not-ground-based experiments on space agriculture have been conducted through the NASA Vegetable Production System (Veggie) and the Advanced Plant Habitat (APH), that grew few species among which edible and ornamental varieties that could be accommodated in the restricted available space aboard the ISS and had the possibility to be studied only for food safety and quality aspects. Future experiments on board of spacecraft or stationary bases will possibly confirm the performances of these engineered tomato also in space.

## Data availability statement

The original contributions presented in the study are included in the article/**Supplementary Material**. Further inquiries can be directed to the corresponding author.

## Author contributions

SM: Conceptualization, Data curation, Formal Analysis, Investigation, Methodology, Supervision, Writing – original draft, Writing – review & editing. RP: Data curation, Formal Analysis, Investigation, Methodology, Software, Visualization, Writing – original draft, Writing – review & editing. ElB: Investigation, Writing – review & editing. ID: Data curation, Formal Analysis, Investigation, Methodology, Writing – review & editing. MV: Conceptualization, Writing – review & editing. AD: Conceptualization, Writing – review & editing. LN: Conceptualization, Investigation, Methodology, Writing – review & editing. EuB: Conceptualization, Funding acquisition, Project administration, Resources, Writing – review & editing. AC: Conceptualization, Data curation, Formal Analysis, Investigation, Methodology, Writing – review & editing.
