# Supplementary material for: Effects of gamma radiation on engineered tomato biofortified for space agriculture by morphometry and fluorescence-based indices
Source: Front Plant Sci. 2023 Oct 9;14:1266199. doi: 10.3389/fpls.2023.1266199 (PMC10591191; doi:10.3389/fpls.2023.1266199)
Supplement: Supplementary file 1 [file DataSheet_1.docx]

**EFFECTS OF GAMMA RADIATION ON ENGINEERED TOMATO BIOFORTIFIED FOR SPACE AGRICULTURE BY MORPHOMETRY AND FLUORESCENCE-BASED INDICES**

**Riccardo Pagliarello^1°,2^, Alessia Cemmi^1§^, Elisabetta Bennici^1°^, Ilaria Di Sarcina^1§^, Maria Elena Villani^1°^, Angiola Desiderio^1°^, Luca Nardi^1°^, Eugenio Benvenuto^1°^, Silvia Massa^1°*^**

^1^ENEA, Italian National Agency for New Technologies, Energy and Sustainable Economic Development.

^°^Biotechnology Laboratory, Biotechnology and Agro-Industry Division, Italian National Agency for New Technologies, Energy and Sustainable Economic Development, Casaccia Research Center, Rome, Italy.

^§^Fusion and Nuclear Safety Technologies Department, Casaccia Research Center, Rome, Italy.

^2^University of Tuscia, DAFNE – Department of Agriculture and Forest Sciences, Viterbo, Italy.

***Corresponding Author:**

Silvia Massa, Tel.: +39 06 3048 4052, FAX: +39 06 3048 4808; email: [silvia.massa@enea.it](mailto:silvia.massa@enea.it)

Running title: Gamma radiation effects on anthocyanin-biofortified tomato

**Supplementary Figures**

**
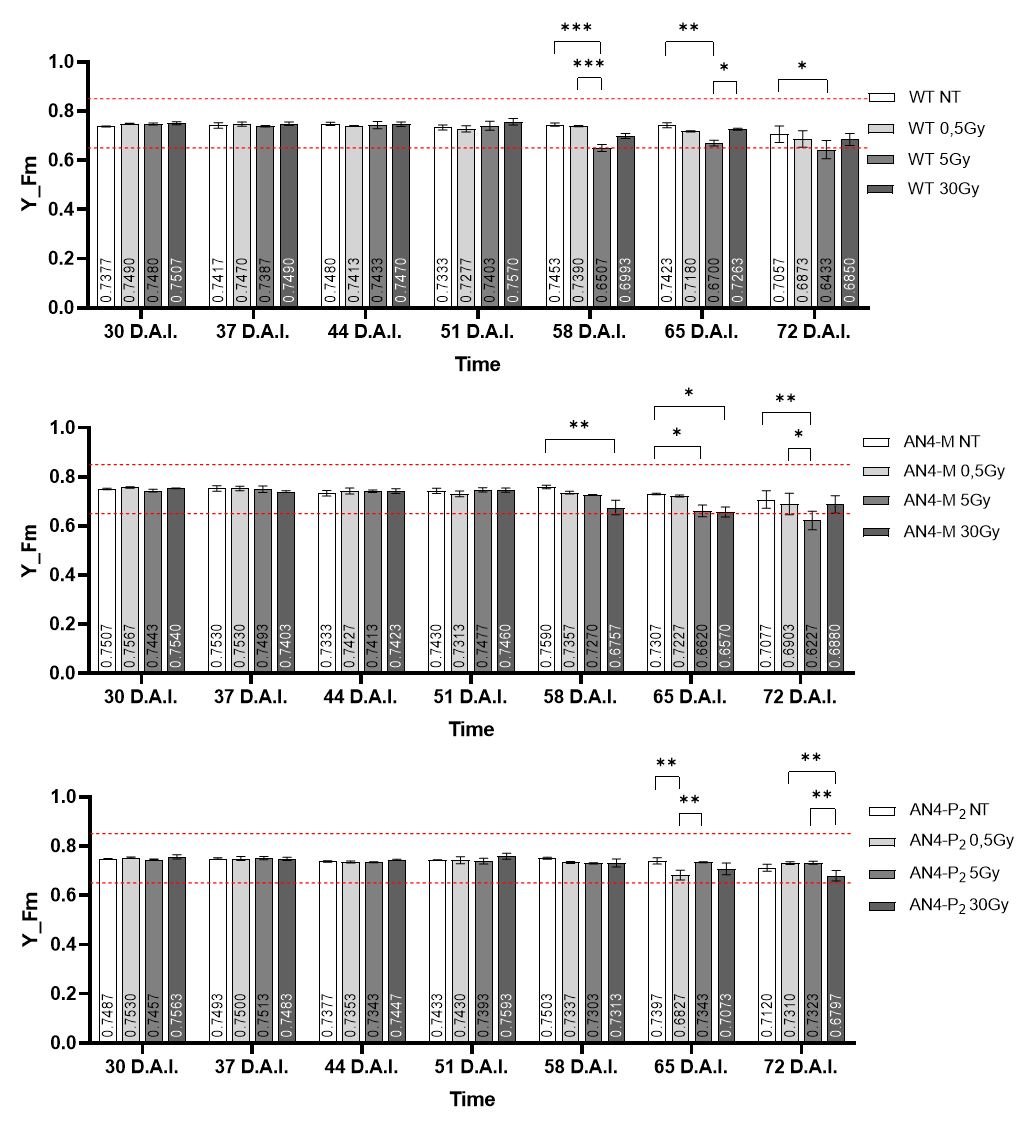
Supplementary figure 1.** *Intra*-genotype analysis of the maximum fluorescence yield index (Y_Fm) of wild type, AN4-M and AN4-P_2_ MicroTom along the observation period upon different irradiation treatments supplied to seeds. Mean values ± SE are shown (𝑛 = 5). Analysis of variance was conducted by two-way ANOVA. Tukey’s post-hoc test was used as a statistical hypothesis testing. Asterisks indicate the statistically significant differences (Statistical relevance: *: p<0.05; **: p<0.001; ***: p<0.0001).

**Supplementary figure 2.** *Intra*-genotype analysis of the flavonols index (FLAV) of wild type, AN4-M and AN4-P_2_ MicroTom along the observation period upon different irradiation treatments supplied to seeds. Mean values ± SE are shown (𝑛 = 5). Analysis of variance was conducted by two-way ANOVA. Tukey’s post-hoc test was used as a statistical hypothesis testing. Asterisks indicate the statistically significant differences (Statistical relevance: *: p<0.05; **: p<0.001; ***: p<0.0001).

**Supplementary tables**

**Supplementary Table 1.** Summary of morphometric and fluorimetric results at the end of the measurement period analysed in comparison within each genotype and according to the different treatments on irradiated seeds. SFR is related to the chlorophyll concentration. NBI is the Nitrogen Balance Index. ANTH is the anthocyanin index. Mean values ± SE are shown (𝑛 = 5). Analysis of variance was conducted by two-way ANOVA with the only exception of fruits-related parameters (one-way ANOVA). Tukey’s post-hoc test was used as a statistical hypothesis testing. Asterisks indicate the statistically significant differences (statistical relevance: *: p<0.05; **: p<0.001; ***: p<0.0001).

|  | | **Irradiation treatments** | | | |  | |
| --- | --- | --- | --- | --- | --- | --- | --- |
| **Genotype** | **Parameter** | **NT** | **0.5 Gy** | **5 Gy** | **30 Gy** | **Legend** | |
| **Wild type** | **Plant height (cm)** | 9.37±0.23 | 8.87±0.42 | 8±0.4 | 9.5±0.28 | + | *** |
|  | **Number of leaves** | 28.75±0.75 | 29±1 | 29.75±0.25 | 29.75±0.25 | + | ** |
|  | **Leaf area (cm^2^)** | 202.54±19.01 | 199.67±5.47 | 214.07±6.16 | 196.48±5.24 | + | * |
|  | **Number of flowers** | 9.5±2.9 | 6.5±1.55 | 14.5±6.39 | 9.25±3.9 | / |  |
|  | **Number of fruits** | 21.25±4.21 | 23.5±3.94 | 18.5±3.37 | 22.5±1.44 | - | * |
|  | **Weight of fruits (g)** | 2.51±0.2 | 2.47±0.2 | 3.1±0.2 | 3.3±0.35 | - | ** |
|  | **Diameter of fruits (mm)** | 13.66±0.46 | 12.6±0.47 | 14.86±0.66 | 15.06±0.88 | - | *** |
|  | **Number of seeds** | 24.6±1.77 | 28±2.04 | 32.66±2.78 | 21±1.7 |  | |
|  | **SFR** | 1.65±0.03 | 1.36±0.12 | 1.51±0.1 | 1.71±0.1 |  |  |
|  | **NBI** | 1.6±0.02 | 1.45±0.05 | 1.45±0.08 | 1.61±0.062 |  |  |
|  | **ANTH** | -0.11±0.003 | -0.11±0.001 | -0.12±0.002 | -0.11±0.001 |  |  |
|  |  |  |  |  |  |  |  |
| **AN4-M** | **Plant height (cm)** | 8.5±0.28 | 8.25±0.49 | 8.75±0.47 | 8±0.4 |  |  |
|  | **Number of leaves** | 23.25±0.47 | 22.5±0.64 | 22.75±0.75 | 22.5±0.64 |  |  |
|  | **Leaf area (cm^2^)** | 152.16±20.07 | 156.97±14.06 | 156.48±15.93 | 171.65±5.24 |  |  |
|  | **Number of flowers** | 3.5±1.25 | 2.25±1.31 | 2±1.22 | 7.5±1.25 |  |  |
|  | **Number of fruits** | 14.75±1.49 | 14.5±2.1 | 20±2.04 | 17.5±0.95 |  |  |
|  | **Weight of fruits (g)** | 2.18±0.21 | 1.98±0.21 | 2.94±0.27 | 3.1±0.22 |  |  |
|  | **Diameter of fruits (mm)** | 12.13±0.6 | 12.66±0.82 | 13.93±0.86 | 16.26±1.78 |  |  |
|  | **Number of seeds** | 20±1.65 | 20±1.49 | 28±1.89 | 32.66±2.16 |  |  |
|  | **SFR** | 1.86±0.1 | 1.58±0.03 | 1.99±0.12 | 1.9±0.09 |  |  |
|  | **NBI** | 1.57±0.07 | 1.35±0.05 | 1.71±0.06 | 1.56±0.02 |  |  |
|  | **ANTH** | -0.09±0.003 | -0.08±0.008 | -0.11±0.002 | -0.11±0.006 |  |  |
|  |  |  |  |  |  |  |  |
| **AN4-P_2_** | **Plant height (cm)** | 8.25±0.74 | 8.25±0.54 | 8.25±0.67 | 8.5±0.64 |  |  |
|  | **Number of leaves** | 18±1.22 | 20.25±0.85 | 17.5±1.7 | 19±1.29 |  |  |
|  | **Leaf area (cm^2^)** | 163.26±17.52 | 171.77±9.54 | 168.45±10.07 | 177.85±13.14 |  |  |
|  | **Number of flowers** | 4.25±1.25 | 7.75±1.65 | 7.75±1.03 | 7.25±1.03 |  |  |
|  | **Number of fruits** | 18.5±1.7 | 14.75±4.49 | 12.75±1.31 | 17.5±2.59 |  |  |
|  | **Weight of fruits (g)** | 3.44±0.22 | 1.99±0.2 | 2.31±0.24 | 2.68±0.27 |  |  |
|  | **Diameter of fruits (mm)** | 15.8±0.48 | 12±0.62 | 14.2±0.88 | 14.13±0.9 |  |  |
|  | **Number of seeds** | 32.66±2.18 | 17.33±1.48 | 20±1.67 | 20±1.38 |  |  |
|  | **SFR** | 1.97±0.08 | 1.7±0.09 | 1.74±0.15 | 1.81±0.13 |  |  |
|  | **NBI** | 1.59±0.02 | 1.55±0.09 | 1.49±0.1 | 1.69±0.1 |  |  |
|  | **ANTH** | -0.08±0.007 | -0.08±0.004 | -0.07±0.01 | -0.086±0.01 |  |  |

**Supplementary Table 2.** Summary of morphometric and fluorimetric results at the end of the measurement period analysed in comparison within each genotype and according to the different treatments supplied to plants at 30 DAS. SFR is related to the chlorophyll concentration. NBI is the Nitrogen Balance Index. ANTH is the anthocyanin index. Mean values ± SE are shown (𝑛 = 5). Analysis of variance was conducted by two-way ANOVA with the only exception of fruits-related parameters (one-way ANOVA). Tukey’s post-hoc test was used as a statistical hypothesis testing. Asterisks indicate the statistically significant differences (Statistical relevance: *: p<0.05; **: p<0.001; ***: p<0.0001).

|  | | **Irradiation treatments** | | | |  | |
| --- | --- | --- | --- | --- | --- | --- | --- |
| **Genotype** | **Parameter** | **NT** | **0.5 Gy** | **5 Gy** | **30 Gy** | **Legend** | |
| **Wild type** | **Plant height (cm)** | 11.56±0.17 | 10.89±0.26 | 11.7±0.59 | 9.69±0.18 | + | *** |
|  | **Number of leaves** | 27.6±0.92 | 33±1.3 | 33.6±1.6 | 28.6±1.16 | + | ** |
|  | **Leaf area (cm^2^)** | 194.65±12.67 | 229.45±14.1 | 219.33±14.98 | 57.05±4.55 | + | * |
|  | **Number of flowers** | 15.4±0.5 | 16.6±0.24 | 17.2±0.66 | 2±0.63 | / |  |
|  | **Number of fruits** | 16.4±1.02 | 16.2±0.37 | 15.4±0.67 | / | - | * |
|  | **Weight of fruits (g)** | 2.84±0.25 | 2.54±0.22 | 2.85±0.23 | / | - | ** |
|  | **Diameter of fruits (mm)** | 17.48±0.68 | 19.88±1.33 | 18.6±0.54 | / | - | *** |
|  | **Number of seeds** | 17.7±1.03 | 21.2±0.75 | 19.7±0.47 | / |  | |
|  | **SFR** | 1.97±0.06 | 2.15±0.06 | 2.23±0.04 | 1.84±0.05 |  |  |
|  | **NBI** | 1.9±0.08 | 1.99±0.01 | 2.16±0.04 | 1.86±0.02 |  |  |
|  | **ANTH** | -0.14±0.008 | -0.12±0.009 | -0.12±0.003 | -0.11±0.001 |  |  |
|  |  |  |  |  |  |  |  |
| **AN4-M** | **Plant height (cm)** | 8.5±0.52 | 9.58±0.13 | 9.3±0.28 | 6.54±0.28 |  |  |
|  | **Number of leaves** | 26±2.12 | 28±2.75 | 27±3.47 | 13±1.22 |  |  |
|  | **Leaf area (cm^2^)** | 116.98±6.79 | 114.5±6.11 | 124.45±7.51 | 83.76±3.49 |  |  |
|  | **Number of flowers** | 18±0.83 | 17.6±0.81 | 18.4±0.6 | 3±0.44 |  |  |
|  | **Number of fruits** | 16.4±0.4 | 16.4±1.2 | 16±0.8 | / |  |  |
|  | **Weight of fruits (g)** | 2.78±0.19 | 2.51±0.17 | 2.3±0.15 | / |  |  |
|  | **Diameter of fruits (mm)** | 16.15±0.57 | 15.89±0.54 | 16.24±0.77 | / |  |  |
|  | **Number of seeds** | 21.5±0.63 | 22.2±0.48 | 21.7±0.3 | / |  |  |
|  | **SFR** | 1.91±0.07 | 2.06±0.05 | 1.8±0.05 | 1.39±0.05 |  |  |
|  | **NBI** | 2±0.04 | 2.07±0.04 | 1.75±0.07 | 1.27±0.07 |  |  |
|  | **ANTH** | -0.09±0.003 | -0.09±0.003 | -0.07±0.004 | -0.07±0.004 |  |  |
|  |  |  |  |  |  |  |  |
| **AN4-P_2_** | **Plant height (cm)** | 9.02±0.32 | 9.74±0.54 | 7.66±0.25 | 7.58±0.19 |  |  |
|  | **Number of leaves** | 18.2±1.8 | 20.6±1.2 | 14.8±1.15 | 10.4±0.5 |  |  |
|  | **Leaf area (cm^2^)** | 114.02±2.3 | 125.49±0.87 | 104.55±5.79 | 68.49±1.42 |  |  |
|  | **Number of flowers** | 19.2±0.37 | 19.2±0.37 | 18.8±0.73 | 2.6±0.5 |  |  |
|  | **Number of fruits** | 15.2±0.37 | 16.4±0.5 | 16±0.54 | / |  |  |
|  | **Weight of fruits (g)** | 2.05±0.26 | 1.61±0.2 | 1.25±0.24 | / |  |  |
|  | **Diameter of fruits (mm)** | 15.29±1.06 | 14.63±0.89 | 13.29±1.46 | / |  |  |
|  | **Number of seeds** | 17.6±0.26 | 20±0.39 | 20.9±0.52 | / |  |  |
|  | **SFR** | 2.1±0.03 | 1.88±0.01 | 2.17±0.1 | 1.58±0.03 |  |  |
|  | **NBI** | 2.2±0.12 | 2.16±0.14 | 2.29±0.15 | 1.61±0.02 |  |  |
|  | **ANTH** | -0.05±0.004 | -0.08±0.009 | -0.06±0.001 | -0.03±0.004 |  |  |
